# Manipulating device-to-body forces in passive exosuit: An experimental investigation on the effect of moment arm orientation using passive back-assist exosuit emulator

**DOI:** 10.1017/wtc.2023.12

**Published:** 2023-05-29

**Authors:** Siddharth Bhardwaj, Akshayraj B. Shinde, Randheer Singh, Vineet Vashista

**Affiliations:** Human-Centered Robotics Lab, Indian Institute of Technology Gandhinagar, Gandhinagar, India

**Keywords:** Design, exosuits, lift assist device, work related musculoskeletal disorders (WMSDs), comfort, biomechanical modeling

## Abstract

Passive exosuits have been vastly researched in the past decade for lifting tasks to alleviate the mechanical loading on the spine and reduce the lower back muscle activities in lifting tasks. Despite promising advantages of exosuits, factors such as comfort directly influence the user’s acceptability of such body-worn devices. Exosuits’ routing/anchoring points, which transmit device-to-body forces, remain the leading cause of discomfort among users. In the present study, we sought to investigate the effect of the routing element, that is, the “moment arm,” in altering the device-to-body forces and perceived discomfort. We first presented a simplified human–exosuit model to establish insight into the effect of the moment arm on the device-to-body forces acting at the shoulder (*F*_S_) and waist (*F*_W_). Further, an experimental investigation was conducted on 10 participants with six different exosuit moment arm configurations (C1, C2, C3, C4, C5, and C6) to investigate their effect on the device-to-body forces, perceived discomfort, and muscle activity using a passive back-assist exosuit emulator in a lifting/lowering task. Configuration C4 was found to be most beneficial in reducing device-to-body forces at the shoulder and waist by up to 44.6 and 22.2%, respectively, during lifting. Subjective scores also comprehended with the device-to-body forces, indicating that C4 produces significantly less discomfort for participants. The outcome of the study illustrates the importance of selecting an appropriate moment arm configuration for passive back support exosuits in alleviating the device-to-body forces and perceived discomfort.

## 1. Introduction

Despite the widespread use of automation and robots in industries, many manual material handling (MMH) tasks involving lifting/lowering, pushing/pulling, and carrying are required to be performed by individuals. It has been estimated that 42% of the EU workers are involved in MMH operations involving carrying/moving heavy loads at least one quarter of the time (Eurofound, 2017). Most often, MMH tasks expose individuals to physical workloads and are aggravated by repetitive movements and awkward postures (Eurofound, 2012), over time leading to work-related musculoskeletal disorders (WMSDs). The U.S. Bureau of Labor Statistics reported over 9,00,000 cases of days away from work (DAFW) in 2018, of which 30% were because of WMSDs alone (U.S. Bureau of Labor Statistics, 2020), placing a substantial burden on the health care system and also leading to a decline in labor productivity (Baldwin, 2004; Coenen et al., 2014). Further, as an estimate, almost 38% of the WMSDs cases in the US relate to either injuries to the lower back or lower back pain (LBP; U.S. Bureau of Labor Statistics, 2018).

Tasks involving heavy and frequent lifting have been shown to be the leading cause of lower back musculoskeletal disorders (LBMSDs) and LBP (Da Costa and Vieira, 2010). Mechanical loading of the lower back during lifting, created by large compressive and shear forces acting at lumbosacral region during lifting, is linked to LBMSDs and LBP (Dolan et al., 1994; McGill, 2007; Bakker et al., 2009). Hence, any intervention created for ergonomic lifting is catered around reducing the spinal loading. For the prevention of LBMSDs several techniques and protocols are being devised – instructing lifting techniques, awareness on WMSDs, exercises for strengthening the lower back, online postural assessments, job redesign, workplace redesign, and following recommended weight lifting standards (Alemi, 2019). However, few approaches render being infeasible and expensive, new directions have been explored in the direction of wearable assistive devices to aid lifting tasks (Ali et al., 2021).

Back support wearable devices for assisting lifting tasks are derived from the human–robot collaboration, involving the use of robotics while retaining human agility. These wearable devices in the form of rigid “exoskeletons” or soft “exosuits” provide an edge in dynamic environments that require human observations and decisions (de Looze et al., 2016). While rigid exoskeletons have the advantage of providing precisely measured assistive torques, soft exosuits have the advantage of being compliant, compact, and less expensive. Moreover, regarding the type of actuation, both active and passive exosuits have been developed in the past for industrial and professional use (de Looze et al., 2016; Toxiri et al., 2019). However, in the current scenario, with active devices being limited by available actuator options, multi-faceted control schemes and power supplies (Wolff et al., 2014; Christensen et al., 2021), passive devices have attracted particular attention in terms of practical utility. These reasons make passive exosuits a more practical solution for load lifting applications (Bosch et al., 2016; Goršič et al., 2021).

A passive exosuit uses elastic bands, gas or torsional springs, or flexible beams with the ability to store energy from human movements and release it when required (Ali et al., 2021). Most of the passive exosuit designed for back support utilizes an elastic element that runs parallel to the spine and thighs; thereby storing the energy in the flexion phase of lifting, which is then released during the extension (upward) phase. The principle has been applied in various passive exosuits, such as PLAD (Abdoli-Eramaki et al., 2007), APEX (Goršič et al., 2021; “HeroWear: Back-assist Wearable Tech for Men & Women | Exoskeleton Technology From HeroWear” n.d.), WAD (Heydari et al., 2013), LiftSuit (“LiftSuit | Auxivo” n.d.), and IPWE (Zeng et al., 2021). Further, the developed exosuits have also shown sufficient efficacy in reducing lumber loading thereby attracting the stakeholders. Studies have reported reduction of 38, 23, and 15% in erecter spine muscle activity while using PLAD (Frost et al., 2009), WAD (Heydari et al., 2013), and APEX (Goršič et al., 2021), respectively. Despite the scientific consensus that exosuits can help reduce muscle activity, fatigue, and the overall physical demand of the task, their use in industries is limited due to design, discomfort, and weight issues (Wolff et al., 2014; Baltrusch et al., 2020), which in turn also influence the user acceptability (Abdoli-Eramaki et al., 2007; de Looze et al., 2016).

Exosuit’s routing elements that transmit the device-to-body forces remain the leading cause of local discomfort at physical human interfaces (Yandell et al., 2020) and are often considered a risk factor for pressure-related tissue injuries (Kermavnar et al., 2021). Hence, careful consideration should be given in designing passive back support exosuits to minimize the perceived forces (device-to-body forces) and retaining them within the comfort limit of the user while lifting (Lamers and Zelik, 2021), using a mathematical model, demonstrated that by introducing an extended moment arm (protruded arm extending from lumbosacral joint) the assistive torque at lumbosacral joint (L5-S1) and device-to-body forces can be modulated. With an extended moment arm, it was shown that similar assistive torque can be generated while reducing the tension in the routed elements, thereby reducing the device-to-body forces. A similar concept was previously adopted in the design of PLAD where the elastic bands were routed over extended pulleys (mounted on hip belt) to generate assistive moment (Abdoli-Eramaki et al., 2007). However, the introduction of an extended moment arm creates an additional device-to-body force at the contact site, which forms another source of discomfort. Therefore, it is essential to consider the device-to-body forces and user comfort at the design step itself to achieve for choosing an appropriate orientation/configuration of a moment arm for passive exosuits.

In the present article, we address the issue of discomfort in passive exosuit by incorporating the device-to-body force in the design step to choose an appropriate orientation/configuration of a moment arm. We first present a mathematical model for gaining insight on the effect of moment arm configurations on device-to-body forces, followed by the design details of the developed passive back-assist exosuit (BASE) emulator for experimentation. Later, we present a lab-controlled experiment using the passive BASE emulator to evaluate the effect of different moment arm configurations on device-to-body forces, perceived discomfort, and electromyography (EMG) in lifting/lowering task.

## 2. Human–exosuit modeling

**Figure 1.**
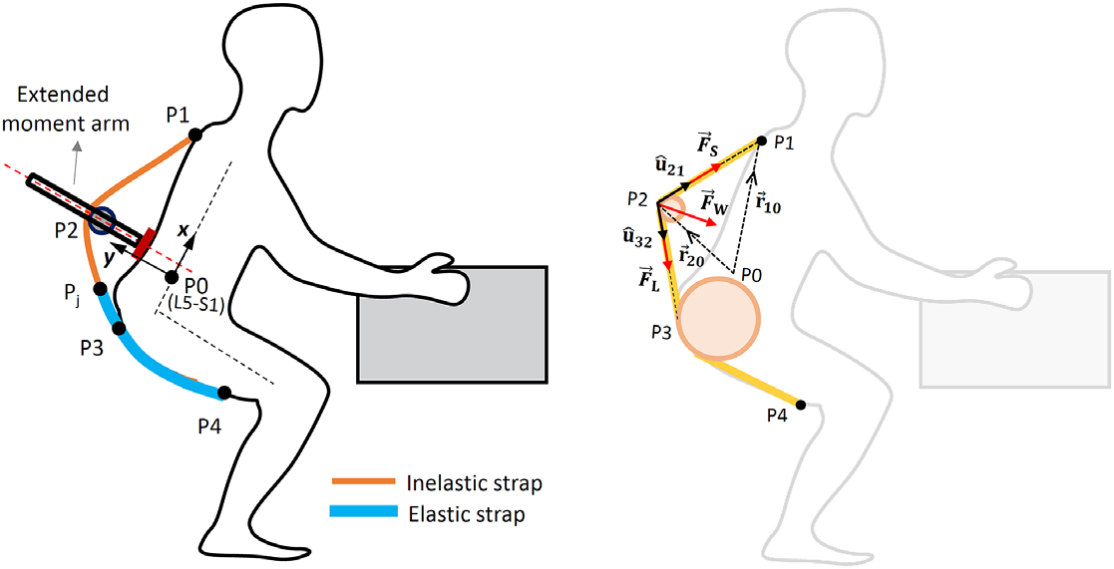
Human–exosuit biomechanical model for estimating the effect of moment arm on the device-to-body forces. The model considers an inelastic strap routed through the shoulder at point P1 and extended moment arm at P2. Elastic strap is attached to the thighs at point P4, leaves the surface contact from the buttocks at P3 and joins the inelastic strap routed through points P1 and P2 at point Pj above the buttocks. 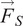 , 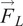 , and 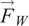 are the device-to-body forces acting at the shoulder, thigh, and waist, respectively, due to stretching of the elastic strap. With the assumption of negligible friction, the tension throughout the routing strap (elastic and inelastic) is modeled as constant, that is, 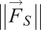 = 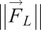 .

Various physiological and biomechanical understanding have been developed in the past to understand the effect of exosuits in offloading lumber spine and associated muscles (Abdoli-Eramaki et al., 2007; Toxiri et al., 2015; Lamers et al., 2018). However, with the adoption of these exosuits in industries, concerns have also been brought about the external forces (device-to-body forces) posed by the exosuits on the human body at the human–exosuit interface. Hence, the need to bring the understanding of device-to-body forces becomes apparent to inform the exosuit design during the prototyping stages. Recently, Lamers and Zelik (2021) provided a simplified human–exosuit biomechanical model to bring a qualitative understanding of how the different exosuits parameters (e.g., the body anchoring points P1, P2, P3, and P4, as shown in Figure 1) affects the device-to-body forces. We have adopted a similar approach in developing a conceptual framework for the present study and modeling the device-to-body forces during load lifting. A static human–exosuit biomechanical model for symmetric sagittal plane lifting was developed considering the device-to-body forces ( 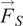 , 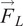 , and 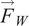 ) due to the routed elastic and inelastic straps, which develop the assistive torque ( 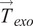 ) at L5-S1 joint (point P0), as shown in Figure 1.

Since, the highest moment is created along the spine at L5-S1 during lifting, the assistive torque ( 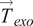 ) was also assumed to be generated around L5-S1 (Bogduk and Macintosh, 1984; Lamers et al., 2018; Lamers and Zelik, 2021). The model considers an inelastic strap routed from point P1 at the shoulder, passing through a frictionless pulley mounted on the moment arm at point P2 and joined to the elastic strap at point Pj (junction of the elastic and nonelastic bands) above the buttocks, as shown in Figure 1. The elastic band is attached to the thighs at point P4 and leaves contact with the body at the lower waist at point P3. Routing the elastic band over the buttocks reduces the relative motion between the band and the buttocks, making it easier for the user to bend and lift. Furthermore, negligible relative motion between the elastic band and the buttocks reduces friction force at the buttocks (Lamers and Zelik, 2021). For this reason, the dissipation work and frictional force at the buttocks are not considered in the presented human–exosuit model.

As depicted in Figure 1, the routed elastic and inelastic straps produce device-to-body forces acting at the shoulder ( 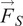 ), thighs ( 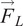 ), and waist ( 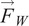 ). Tensile forces 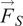 and 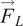 act at the point P1 and P4, respectively, due to tension in the routed elastic/inelastic strap. While a compressive force, 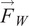 , acts at the waist along the moment arm. From the force triangle in Figure 1, the device-to-body forces are related as in Eq. (1). Where 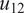 and 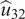 are the unit vectors along the tension forces between points P2–P1 and P3–P2, respectively.
(1)

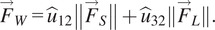

The device-to-body forces, 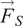 and 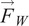 , generate an assistive toque ( 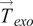 ) at L5-S1 joint (point P0, Figure 1). Since the assistive torque is developed at the trunk (point P0), 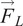 will not contribute to 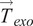 . Expression for the generated assistive torque at L5-S1 by the exosuit can be written in terms of the torque 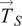 due to tension in the shoulder strap ( 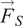 ) and torque 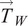 due to force 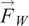 exerted by the extended moment arm on the waist as is given in Eq. (2). From the free-body diagram shown in Figure 1, after a few algebraic manipulations and using Eq. (1), the torque components, 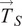 and 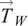 , are written in terms of 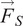 and 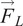 as in Eq. (3), where 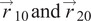 define the vectors 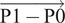 and 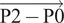 , respectively, and 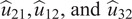 represent the unit vectors to define forces in the direction of 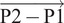 , 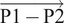 , and 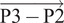 , respectively, as depicted in Figure 1. Further, since friction is not considered in the model, the tension magnitude in elastic/inelastic strap can be assumed to be the consistent between routing points P1–P4, such that, 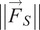 = 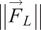 . Thereby, the expression for 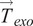 is simplified as in Eq. (4).
(2)

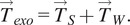



(3)

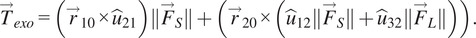



(4)

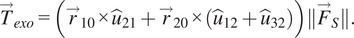

For a fixed shoulder point P1, Eq. (4) implies that 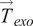 is dependent on the configuration of the extended moment arm (i.e., the location of point P2). We can therefore define the effective moment arm of the exosuit, 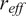 (perpendicular distance between the force 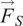 and point P0), for the generated assistive torque 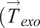 ) as in Eq. (5).
(5)

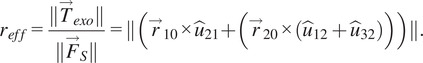

Also, from the Eq. (1) and no friction assumption ( 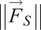 *=* 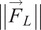 ), 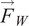 can be written as in Eq. (6). Further, considering the magnitude of forces, Eq. (6) can be written as in Eq. (7). Where 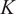 = 
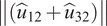
 is the ratio between the magnitude of device-to-body force at the waist ( 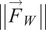 ) and shoulder ( 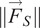 ).
(6)

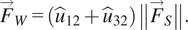



(7)

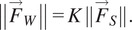

From Eq. (5), it is observed that for a particular amount of 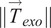 a larger moment arm results in lesser shoulder force 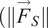 ). However, as evident from Eq. (7), this will also influence the compressive force at the waist ( 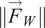 ) based upon the factor *K.* In a nutshell, these equations suggest that for fixed body anchoring points (P1 and P3), consideration should be given to the location of routing point P2 to manipulate device-to-body forces. Hence, the configuration of the extended moment arm must be such that it can increase the 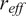 but reduces the device-to-body forces posed by the exosuit. To observe this variation in device-to-body forces and assistive torque, a model exploration was conducted for different locations of point P2 (*x*2, *y*2). Location for points P1 and P3, representing the shoulder anchoring point and buttocks contact point, were approximated based upon the average measurement from a male participant representing 50 percentile Indian male population (≈170 mm stature; Chakrabarti, 1997); origin P0(*x*0, *y*0) = (0, 0); P1(*x*1, *y*1) = (0.38 m, 0.08 m); and P3(*x*3, *y*3) = (−0.15 m, 0.05 m). The simulation results depicting the variation in factor *K* and 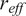 are presented in section 4.2.

Although the model presented here suggests that device-to-body forces can be manipulated by changing the moment arm orientation. But for the fabric based exosuits, analytical evaluation of device-to-body forces becomes difficult due to multiple device-body contact points. Hence, an experimental evaluation of device-to-body forces at the shoulder and waist should be conducted before the prototyping stage. In the present work, we experimentally investigated the effect of different configurations of the moment arm on the device-to-body forces, perceived discomfort and muscle activity using a passive BASE emulator (Bhardwaj et al., 2022). The emulator provides the distinctive advantage of having an extended modular moment arm that can be configured to test various moment arm configurations. Six potential configurations of the moment arm (C1–C6, as described in section 4.2). were chosen to investigate its effect on the EMG, device-to-body forces and perceived discomfort. The emulator design and experiments are described in the following sections.

## 3. Passive back-assist exosuit emulator

### 3.1. Mechanical design

**Figure 2.**
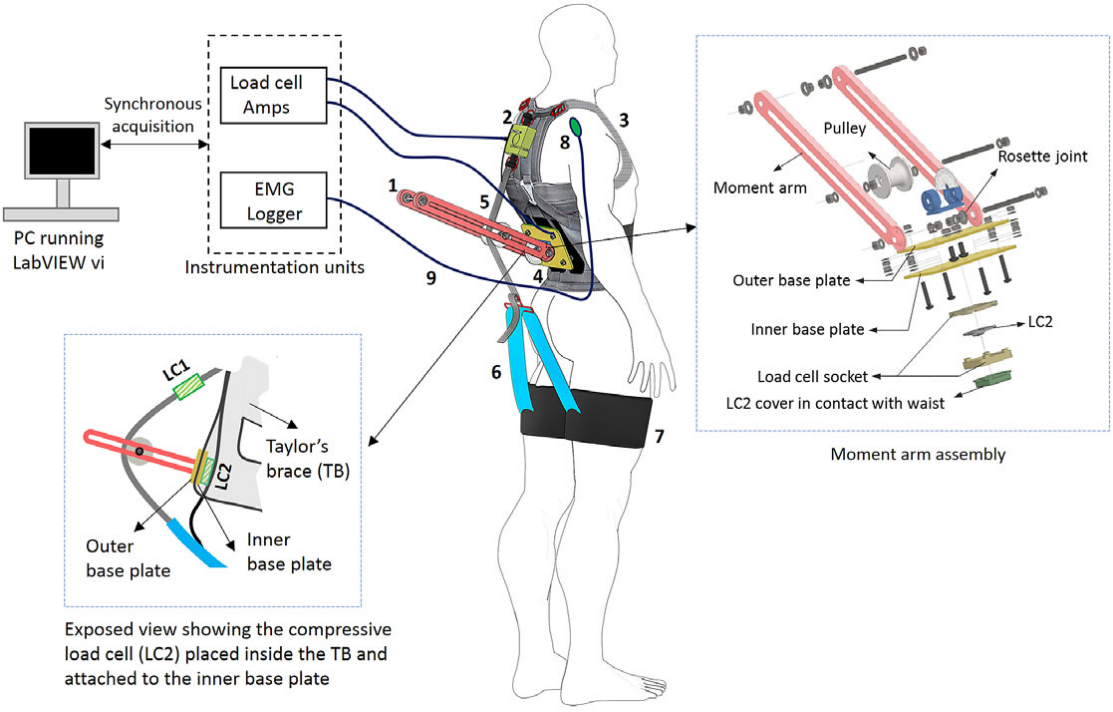
Schematic of the passive BASE emulator (Bhardwaj et al., 2022) with instrumentation system showing: (1) moment arm, (2) tensile load cell (LC1), (3) Taylor’s Brace (TB), (4) outer base plate for mounting moment arm, (5) nylon strap, (6) elastic strap, (7) thigh cuff, (8) surface EMG electrode, and (9) wired connection to instrumentation systems.

The designed emulator comprises of a wearable fabric brace, a passive actuation element (elastic strap) and an extended moment arm, as depicted in Figure 2. The upper body elasto-fabric brace comprised of a Taylor’s Brace (TB) to support the trunk. While a thigh-cuff wound at the mid thighs forms the lower segment of the passive BASE emulator. An elastic strap with a stiffness of 700 N/m was routed from the thigh cuffs and joined to the inelastic nylon strap using a length-adjustable slide buckle, ensuring conformity with different users. The elastic strap was routed over the buttocks and stretches once the user squats or bends, thereby generating an assistive torque in load lifting/lowering activities. With the existing strap routing, the spinal load during lifting task was distributed between the trunk, shoulders, and thighs. The moment arm assembly comprises the extended leg (rails) and carries the pulley, which can be translated posteriorly to change the effective length of the moment arm. The moment arm assembly is mounted on the TB using inner and outer base plates such that the TB is sandwiched between the two base plates. The outer base holds the moment arm legs with the help of a rosette joint, allowing to position the moment arm in different orientations. In addition, a provision has been made in the TB to translate the moment arm assembly vertically up or down along the spine, ensuring that the base of the moment arm can be placed at L5-S1 joint irrespective of user anthropometry. Further details on the design of passive BASE emulator are provided in Bhardwaj et al. (2022).

### 3.2. Instrumentation

Device-to-body forces at the shoulder and waist were acquired using separate uniaxial load cells. A tensile load cell (LC1), placed in tandem with the nylon strap, measures the device-to-body force at the shoulder (*F_S_*). While a compression load cell (LC2) was mounted on the inner baseplate and positioned over the L5-S1 joint to measure the normal force posed by the moment arm at the waist (*F_W_*). Load cells were pre-amplified using HX711 24-bit ADC and connected to data acquisition PC using Arduino Uno (ATmega328), sampled at 90 Hz. In addition, eight-channel EMG system (MWX8, Biometrics Ltd., UK) with bipolar surface EMG electrodes (SX230, Biometrics Ltd., Newport, UK) was used to capture the muscle activities. Each EMG channel was sampled at 1,000 Hz. Both EMG and load cell data were acquired synchronously via custom build LabVIEW vi. Figure 2 illustrates the sensorial interfacing.

## 4. Experiment

### 4.1. Participants

Ten healthy male participants (age: 21–28 years, height: 171.14 ± 9.37 cm and weight: 71.24 ± 5.16 kg) with normal spine participated in the study. All the participants had no history of musculoskeletal disorders in the past 6 months. Prior to conducting the experiment, participants were told about the experiment and signed informed consent was obtained as per the institute ethics committee (study approval no. IEC/VV/2021/013).

### 4.2. Experiment design and procedure

**Figure 3.**
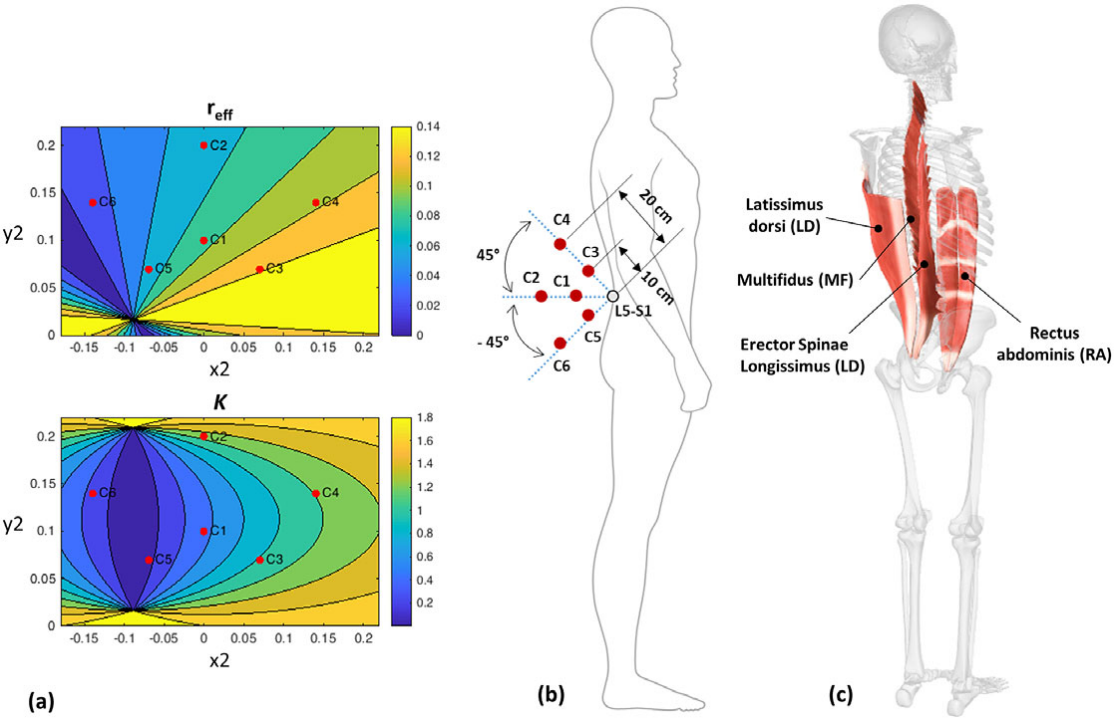
(a) Simulation results based on the developed model showing the effect of different moment arm locations (point P2 (*x*2, *y*2), Figure 1) on the force ratio *K* and effective moment arm *r_eff_*, for the designed emulator. The red dots depict the chosen six configurations such that each dot caters for the different regions of the contour plot. (b) Different exosuit moment arm configurations, C1–C6 chosen for the experimental investigation. (c) Upper body muscles considered in the study.

A full factorial design was directed for the experiment, where each participant performed the lifting/lowering task with a weighted crate for the seven different experimental conditions (C1, C2, C3, C4, C5, C6, and NoExo), repeated twice. Lifting weight was set to 20% of the participant’s body weight. The moment arm configurations, C1–C6, were performed while wearing the passive BASE emulator and symbolize the six different moment arm configurations, as described in Figure 3b. These chosen exosuit configurations catered the three distinct regions: above (C3 and C4), inline (C1 and C2), and below (C5 and C6) the L5-S1 joint. Configurations C1, C3, and C5 were at a radially shorter distance from L5-S1 than configurations C2, C4, and C6. The configurations (C1–C6) were selected based upon the simulation results obtained using Eqs. (4) and (6) such that the configurations C1–C6 represent different values of *r_eff_* and *K*, as shown in Figure 3a. Further, for easier implementation, the specific choice (out of several) of the six potential tested configurations was made such that the sagittal symmetry of the chosen six configurations of the moment arm was maintained. NoExo referred to the experimental condition when lifting/lowering was performed without wearing passive BASE emulator. Conditions were randomized using a 7 × 7 Latin square design to ensure that no participant had the same sequence of conditions for the experimental task. During the experiment, EMG activities of both left and right segment muscles (as shown in Figure 3c), namely, multifidus (MF), longissimus segment of erector spinae (ES), latissimus dorsi (LD), and rectus abdominis (RA); device-to-body forces at the waist (*F_W_*) and shoulder (*F_S_*); and subjective discomfort scores were recorded, thereby forming the dependent variables of the study. While the different experimental conditions form the independent variables of the study.

**Figure 4.**
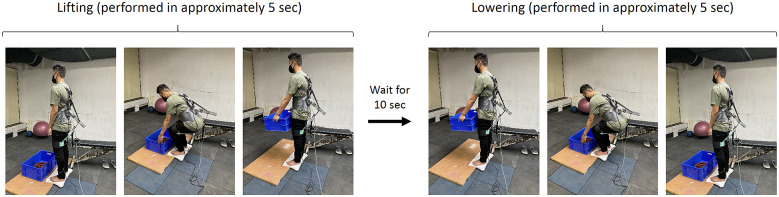
Study protocol: A participant first lift the loaded crate, waits 10 s with the loaded crate in upright posture and then lowered the crate. The cadence duration for lifting/lowering was 5 s.

The experimental task consisted of performing a single lifting/lowering cycle – Starting from the standing posture, the participant first lifted the loaded crate in squatting posture, held it for 10 s while standing and then lowered the crate in squatting posture, as depicted in Figure 4. Each lifting and lowering cadence of the experimental trial was performed in approximately 5 s each. After briefing the participant about the experiment, training trials for squat lifting/lowering were performed to acquaint the participant with the task and task speed using a metronome. Once the participant felt familiarized with the task, EMG sensors were placed on the MF, ES, LD, and RA (both left and right segments). The muscles’ maximal voluntary isometric contraction (MVIC) were recorded by exercising Superman hyperextension for MF and ES (Kim et al., 2016), Lats pull-down for LD (Park and Yoo, 2013) and hollow body hold for RA (Drysdale et al., 2004). The skin preparation and placement of EMG and grounding electrodes were done as per the SENIAM standards (Hermens et al., 2000). After MVIC recordings, actual experimental trials were conducted, separated by a rest period of at least 5 min. During the experimental trials, EMG activities of the above-mentioned muscle groups and force data from the shoulder (LC1) and waist (LC2) load cells were collected. Further, at the end of each trial, participants were asked to report the subjective discomfort using a combination of visual analog scale (VAS) and 27 segments Corlett and Bishop’s localized discomfort map (Nolimo Solman, 2002; Bhardwaj and Khan, 2018).

### 4.3. Data analysis

For post-processing, the acquired EMG was band-passed between 20 and 400 Hz and evaluated for root mean square (RMS) feature using a sliding window of 125 ms for further processing for each muscle during the task. The data for each muscle were further normalized (nRMS) using MVIC method (Burden and Bartlett, 1999), as represented by Eq. (7).
(7)

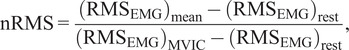

where, RMS, root mean square of raw EMG; MVIC, maximum voluntary isometric contraction; nRMS, MVIC normalized EMG RMS; 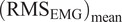 , mean EMG RMS during the task; 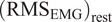 , EMG RMS during rest, computed during relaxed standing posture before MVIC recordings; 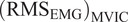 , maximum EMG RMS during isometric muscle contraction exercise.

For device-to-body force data, maximum values for *F_W_* and *F_S_* were obtained during the experimental trials, representing the peak force at the waist and shoulder, respectively. In the analysis of subjective VAS scores, only those segments were considered that were reported by at least two participants. Reported frequencies of the discomfort body part and mean discomfort scores were used for deducing the subjective results of the study. EMG and force data were analyzed separately for lifting and lowering within the 5 s cadence window. However, the subjective ratings were analyzed a single score for combined lifting and lowering.

### 4.4. Statistics

Dependent variables (device-to-body forces, subjective scores, and muscles’ EMG nRMS) were tested for normality using Shapiro–Wilk’s test. Being rejected for normality, the device-to-body forces and subjective scores were tested for any significant difference between experimental conditions (C1, C2, C3, C4, C5, C6, and NoExo) using Kruskal–Wallis nonparametric test. Subsequently, Mann–Whitney pairwise comparison with Bonferroni correction was applied to evaluate group differences (between experimental conditions) for significant Kruskal–Wallis results. Multivariate analysis of variance (MANOVA) was used to test the significance of the tested configurations on individual muscle’s EMG nRMS, and subsequent Tukey’s HSD post hoc test was applied for multiple pairwise comparisons. The level of significance (*p*-value) for all the statistical tests was set to .05.

## 5. Results

**Figure 5.**
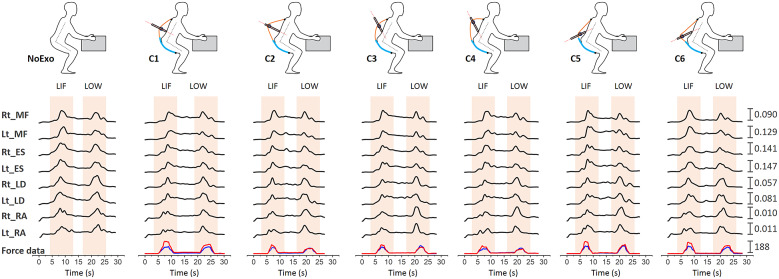
Representative plot for a participant during lifting (LIF)/lowering (LOW) task showing the EMG envelopes (mV) for MF, ES, LD, and RA (right [Rt] and left [Lt]) segments for NoExo, C1, C2, C3, C4, C5, and C6 configurations of moment arm. Force data (N) at the waist, FW (red), and shoulder, FS (blue) are also shown for different moment arm configurations.

Figure 5 shows a representative plot for the EMG and device-to-body forces (*F_W_* and *F_S_*) during the lifting/lowering task with (C1, C2, C3, C4, C5, and C6 configurations) and without (NoExo) the emulator. Visual analysis depicted the difference in peak EMG activity and force levels for the different tested conditions. Compared to the NoExo, using exosuit (in any configuration) was found to reduce the mean EMG activity in all the muscles. The device-to-body force at the shoulder (*F_S_*) and waist (*F_W_*) were found to be different among the tested moment arm configurations. The following subsections detail the results obtained for the device-to-body forces, subjective discomfort rating and EMG activity.

### 5.1. Device-to-body forces

**Figure 6.**
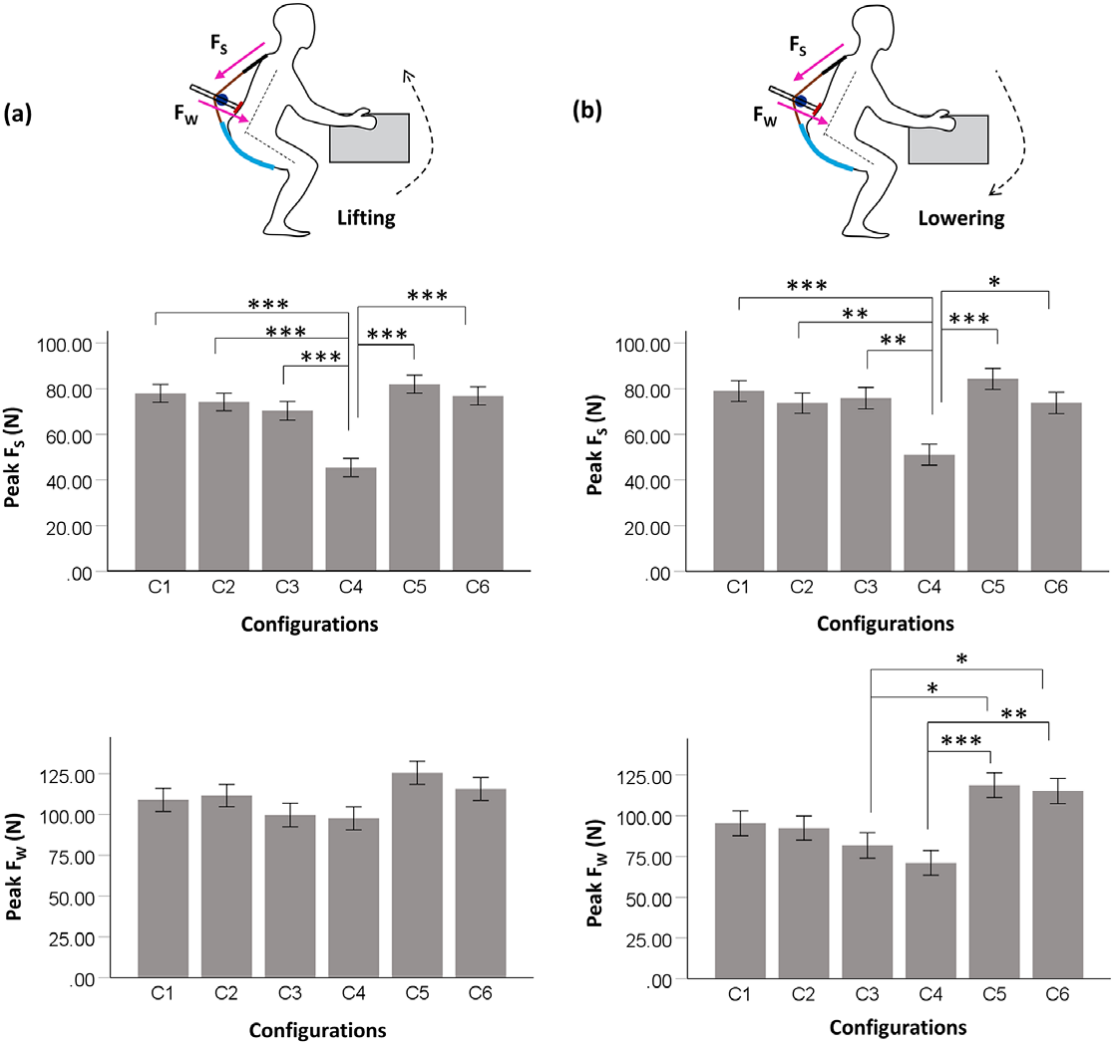
Peak device-to-body forces (mean [SE]) at the shoulder (FS) and waist (FW) during (a) lifting and (b) lowering. Asterisks shows the significant pairs from post hoc test. *p < .05, ** p < .01, *** p < .001.

Figure 6a shows the peak device-to-body forces at the shoulder (*F_S_*) and waist (*F_W_*) during lifting. Kruskal–Wallis test showed that *F_S_* was significantly different across the different moment arm configurations (*p* < .001). Further, post hoc test revealed that C4 configuration was associated with a significantly lower *F_S_* magnitude (45.43 ± 12.29 N) in lifting compared to all other tested configurations (all *p* < .001), as depicted in Figure 6a. For *F_W_*, no significant difference was found among the tested configurations (C1, C2, C3, C4, C5, and C6) during lifting. However, as depicted in Figure 6a, C4 configuration was associated with lower *F_W_* (97.62 ± 33.38 N) compared to other configurations.

During lowering, a trend similar to lifting activity was reported for device-to-body forces between tested configurations (Figure 6b). From Kruskal–Wallis test it was found that both *F_S_* (*p* < .001) and *F_W_* (*p* < .001) were significantly different across the tested configurations. Post hoc analysis showed that during release, *F_S_* was significantly less in C4 configuration (51.11 ± 14.10 N) compared to C1 (*p* < .001), C2 (*p* = .010), C3 (*p* = .004), C5 (*p* < .001), and C6 (*p* = .012). Configuration C4 was also found to have significantly lower *F_W_* (71.08 ± 35.01 N) in lowering compared to C5 (*p* < .001) and C6 (*p* < .002) configurations. In addition, C3 configuration was also found to have significantly lower magnitude of *F_W_* (81.80 ± 27.58 N) compared to C5 (*p* = .015) and C6 (*p* = .048) configurations.

For the experimental task, configuration C5 was associated with highest device-to-body forces during lifting and lowering. While C4 configuration was found to produce least device-to-body forces, reducing *F_S_* and *F_W_* by 44.6 and 22.2%, respectively, in lifting, and 39.3 and 40.1%, respectively, in lowering compared to C5 configuration.

### 5.2. Subjective rating

**Figure 7.**
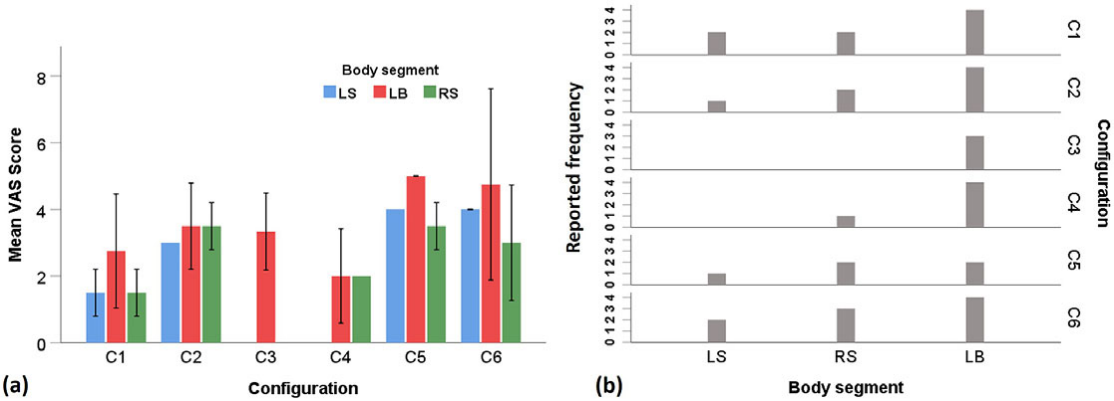
Results for subjective scores showing the (a) VAS rating (mean [SE]) and (b) discomfort reporting frequency as reported by the participants for different body segments in different moment arm configurations. LS, left shoulder, LB, lower back, RS, right shoulder.

Only those body segments which were reported by at least two participants during the whole experiment were considered for the analysis. The results for the subjective rating are summarized in Figure 7. During the experiment, right shoulder (RS), left shoulder (LS), and lower back (LB) were found to be the most reported body segment for discomfort.

Irrespective of the tested emulator configurations, LB was found to be the most reported discomfort site by the participants during the task (Figure 7b). Although no significant difference was found from Kruskal–Wallis test for the effect of configuration on the VAS score, it was observed that C4 configuration caused least LB discomfort compared to other configurations (Figure 7a). For LB the reported discomfort (mean) for different configurations were: C4 (2.00) < C1 (2.75) < C3 (3.33) < C2 (3.50) < C6 (4.75) < C6 (5.00). Concerning shoulder discomfort, none of the participants reported discomfort in LS and RS in configuration C3, while only one participant responded discomfort in RS for C4 configuration. In all other configurations, shoulders (both LS and RS) were found to be reported more frequently by the participants for discomfort, as depicted in Figure 7b. For LS and RS, the mean reported VAS scores for different configurations were C1 (1.50) < C2 (3.00) < C5 (4.00) = C6 (4.00) and C1 (1.50) < C4 (2.00) < C6 (3.00) < C2 (3.50) = C5 (3.50), respectively.

### 5.3. Electromyography

While analyzing the EMG activities of individual participants, it was observed that three out of 10 participants showed a different trend in EMG activities among the tested conditions. The three participants showed an increased EMG activity in most of the muscles for lifting/lowering with exosuit (configuration C1, C2, C3, C4, C5, and C6) compared to NoExo. For statistical analysis on EMG data, these three participants were excluded. However, a general discussion has been made about the reported differences in EMG activities of excluded participants in the discussion section.

**Figure 8.**
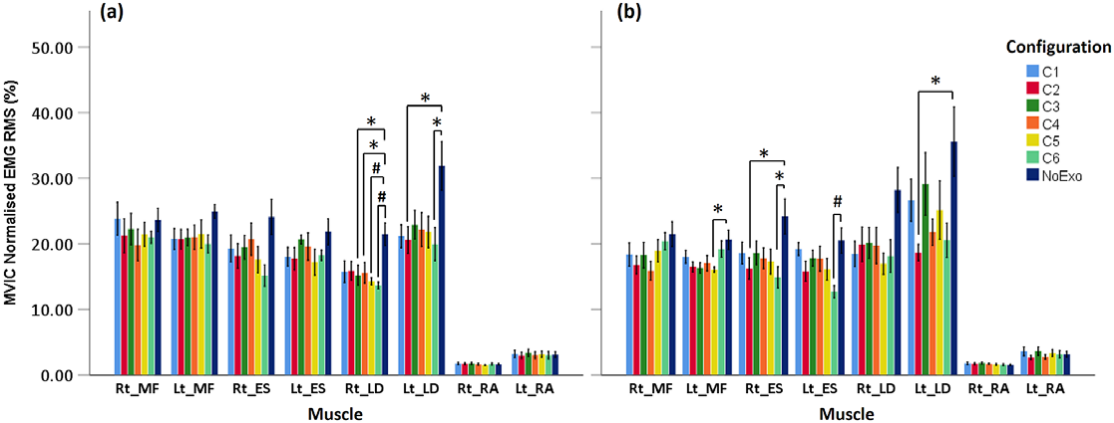
Normalized EMG RMS (mean [SE]) for MF, ES, LD, and RA muscles (both left [Lt] and right [Rt] segments) during (a) lifting and (b) lowering activity for different experimental configurations for seven participants which showed reduction in EMG activities with exosuit compared to NoExo. MF, multifidus, ES, erector spinae, LD, latissimus dorsi, RA, rectus abdominis. *p < .05 and #p < .01 show the significant pairs from post hoc analysis.

Figure 8 shows the normalized EMG RMS (nRMS) amplitudes for lifting and lowering. Visual inspection of the data shows that for the majority of muscles considered in the study, the nRMS was higher for NoExo compared to different exosuit configurations for both lifting and lowering. For lifting, MANOVA revealed that nRMS of Rt_LD (*p* = .004) and Lt_LD (*p* = .021) were significantly different across the tested configurations. Further, Tukey’s pair wise comparison showed that during lifting, nRMS of Rt_LD was significantly lower for C6 (*p =* .002), C5 (*p =* .007), C4 (*p =* .048), and C3 (*p =* .029) configurations compared to NoExo. While nRMS of Lt_LD was found to be significantly lower for C6 (*p =* .021), and C2 (*p =* .030) configurations than NoExo.

For lowering, MANOVA showed significant difference in nRMS of Lt_MF (*p* = .018), Rt_ES (*p* = .031), Lt_ES (*p* = .019), and Lt_LD (*p* = .026). Post hoc test revealed that Lt_MF nRMS was significantly lower for configuration C5 compared to NoExo (*p* = .037); nRMS of Rt_ES was significantly lower for C6 (*p* = .016) and C2 (*p* = .048) compared to NoExo; nRMS of Lt_ES was significantly lower for C6 (*p* = .010) compared to NoExo; and nRMS of Lt_LD was significantly lower for C2 (*p* = .020) compared to NoExo. However, no significant difference in nRMS was observed among the different moment arm configurations (C1, C2, C3, C4, C5, and C6) for both lifting and lowering.

## 6. Discussion

Various passive exosuit design implementations have been explored in past literature for assisting the lower back during lifting/lowering tasks (Abdoli-Eramaki et al., 2007; Heydari et al., 2013; Zeng et al., 2021). However, comfort remains a dominant factor in the overall acceptance of these exosuits irrespective of the proven reduction in spinal loading (Abdoli-Eramaki et al., 2007; Wolff et al., 2014; de Looze et al., 2016; Baltrusch et al., 2020). Pressure points created by the device-to-body forces remain an important question to address while designing such exosuits and are the foremost reason for creating discomfort to the user (Yandell et al., 2020; Kermavnar et al., 2021). The study evaluated the effect of moment arm configuration in passive exosuit designs during lifting/lowering tasks. Six different moment arm configurations were tested for their effect on the device-to-body forces, perceived discomfort, and EMG activities of the back and trunk muscles using the passive BASE emulator. The results indicate that the configuration of the moment arm significantly affects the device-to-body forces and perceived discomfort. However, no significant difference was observed in the EMG activities of the lower back, upper back, and trunk muscles recorded during lifting/lowering activities with different moment arm configurations. The study indicates the importance of selecting an appropriate moment arm of passive back support exosuits in alleviating the device-to-body forces and perceived discomfort.

### 6.1. Effect of moment arm

Back extensors muscles and related passive tissues are responsible for creating the “internal” extension moment that supports the torso during the upward phase of lifting/lowering activities. However, these muscles and ligaments act at very small moment arms (Nemeth and Ohlsen, 1986) than the CG of the upper body, causing them to generate large forces which not only cause fatigue but also add compressive forces to the lumbar spine (Hoozemans et al., 2008). In passive exosuit designs, a similar concept is being utilized where the routing straps/fabric runs parallel to the lower back muscles and creates an “external” assistive moment that reduces the effort from the lumbar spine (Abdoli-Eramaki et al., 2007; Lamers et al., 2018). Being positioned outside the human body, the exosuits have the advantage of designing an extended moment arm that can further improve the external torque. However, its relation to device-to-body forces has not been evaluated experimentally in the past. Simulation results from a simplified human–exosuit model (Figure 3a) show that the choice of location of point P2 (moment arm configuration) can influence the device-to-body forces. Although outside the scope of this study, other body-exoskeleton anchor points (P1, P3, and P4) have also been shown to affect the device-to-body forces (Lamers and Zelik, 2021).

Among the six tested moment arm configurations, configuration C4 was found to generate the least device-to-body forces at the shoulder and waist during lifting/lowering. Subjective scores supported this observation, and it was found that participants reported least discomfort in configuration C4 during lifting/lowering. For all the tested moment arm configurations, a considerable number of participants reported discomfort in the lower back (LB), which could be attributed to the force generated at the waist during the task. Further, the small surface area of the load cell (LC2) in contact with the spine has also increased the localized pressure giving the feeling of discomfort to the participants. This can be further reduced by redesigning the exosuit’s lumbar interface to distribute the generated force at the waist, such as through the posterior support cushions or other soft interfaces (Chang et al., 2020).

Despite concerns on the discomfort scores, all the participants reported that they felt some assistance while using the passive BASE emulator irrespective of the moment arm configuration. From EMG analysis, it was evident that there was a reduction in nRMS of back muscles for all the tested moment arm configurations compared to NoExo. However, no significant difference in EMG nRMS was reported across the tested moment arm configurations (C1–C6). Hence, it becomes evident that for a given assistive torque, moment arm configuration affects the device-to-body forces (Lamers and Zelik, 2021) but does not affect the EMG activities of the monitored muscle group. It was interesting to observe that RA muscle activity remained similar whether or not the exosuit was used. Similar observation was reported by Bosch et al. (2016) while evaluating the effect of passive Laevo exoskeleton in forward bending task, where the normalized EMG amplitude of the obliquus abdominus and rectus abdominus showed no significant difference with and without the exoskeleton. However, literatures are also available where both increase (Alemi et al., 2019) and decrease (Koopman et al., 2019) in EMG amplitude of the abdominal muscle have been reported with the use of back support devices. In addition, intra-abdominal pressure associated with abdominal muscle activation has been shown to favor spinal unloading during trunk extension, that is, the ascending phase of lifting tasks (Essendrop et al., 2002; Stokes et al., 2010). This distinctive trend in abdominal muscle activities with the use of back-assistive devices indicates the possibility of interaction between the exosuit belts forming a corset around the torso on the intra-abdominal pressure, which needs further exploration.

**Figure 9.**
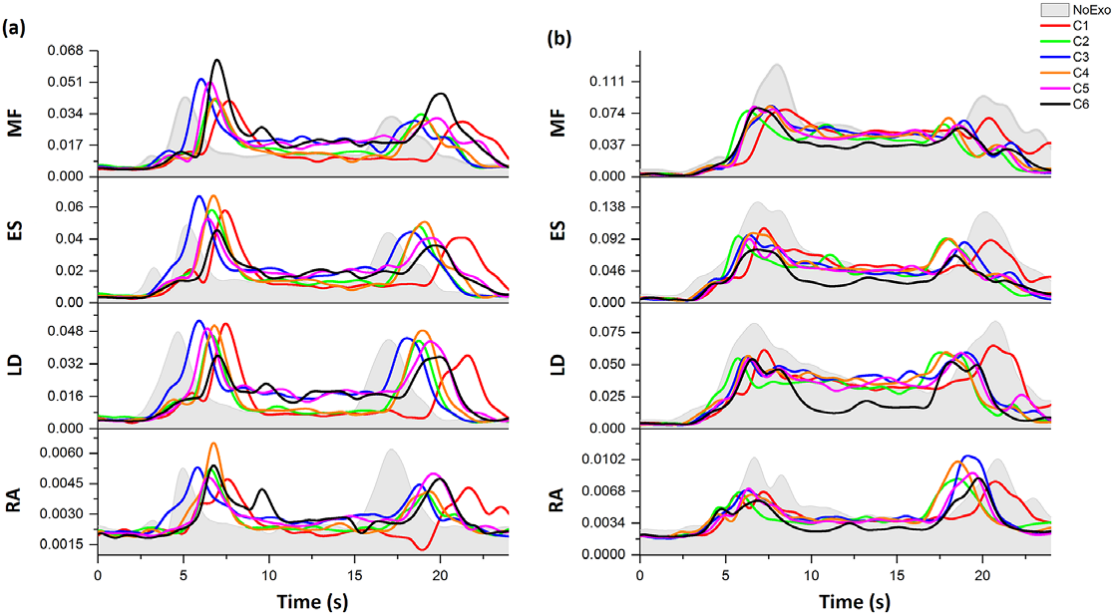
EMG envelopes (mV) depicting the two different patterns of muscle activation observed for the participants during the lifting/lowering task. (a) Representative plot for a participant showing an increase in peak EMG activity of MF, ES, LD, and RA while wearing exosuit (for C1–C6 configurations) compared to NoExo condition (shown by grey filled curve). Such EMG behavior was present in three out of 10 recruited participants. (b) Representative plot for a participant showing a decrease in muscles’ peak EMG with exosuit.

Interestingly, during the EMG analysis, we found three out of 10 subjects whose muscle activity was higher while performing the task with exosuit (in C1–C6 configurations) compared to NoExo condition. This was peculiar since all other subjects showed a trend of reducing muscle activity while performing the task wearing exosuit. Figure 9 shows a sample plot depicting the two distinct trends observed among the participants. As depicted by the representative plot in Figure 9a, few participants showed an increase in muscles’ EMG amplitude with exosuit compared to NoExo condition. While other participants showed a reduction in EMG amplitude with the use of exosuit, indicating the assistance provided by the exosuit in lifting/lowering task, as shown in Figure 9b. The two different trends in EMG activation observed among the participants can be attributed to the fact that few participants co-contracted their muscles during the task compared to letting the exoskeleton support the upper body (Alemi et al., 2019). Although we are short of such data and the reason causing the participants to increase the muscle activity with exosuit usage, an introductory training to the workers on using exosuits should be considered for reducing the joint loading as excessive co-contraction has been significantly correlated with the LBP (Schinkel-Ivy et al., 2013).

### 6.2. Practical implication

Distinctive to form-fitting passive exosuits (Lamers et al., 2018), exosuits with extended moment arm can provide users with a way to increase the assistive torque, which is required in scenarios where heavy loads are being lifted. However, a configuration of moment arm for a specific assistive torque can be chosen, which can result in decreasing the device-to-body forces. As presented in the study, careful consideration of the exosuit moment arm can significantly impact the user’s comfort while using passive exosuits for lifting/lowering. Whereas many passive back support exosuits have been designed in past, the framework of the current study can be adopted to design the extended moment arms for different MMH applications.

Another implication of this study is the design of the passive BASE emulator, which can be used to experimentally test the effect of various human–exosuit anchoring points on biomechanical loads, ergonomics and assistance levels during MMH applications. We have seen various active emulator systems that are used to practically test multiple aspects of design and control schemes (Chiu et al., 2020; Bryan et al., 2021). However, to date, we haven’t come across any emulator system for passive exosuit that could be used to test different design aspects before prototyping. With the aid of such an emulator, researchers and designers can readily experiment to test various design configurations, routing points, anchoring points, and elastic member stiffness levels for informed design development and prototyping.

### 6.3. Limitations and future scope

We adopted a simplified modeling approach to establish an understanding on the device-to-body forces exerted by the passive exosuit neglecting the spine and soft-body mechanics. However, the model was adequate to provide a general understanding of the effect of the moment arm on device-to-body forces. Further, the presented lab-controlled study only included symmetric (sagittal plane) lifting/lowering tasks at a controlled cadence on male participants. However, in actual work conditions concerning MMH, the workers are exposed to varying tasks, for example, walking while holding the load, maintaining static postures, performing stoop lifting, etc., and require separate consideration, especially when evaluating a specific exosuit design. Further, as a limitation, we did not consider the participant’s personal factor and handle height during lifting, whose effect needs separate consideration. As a future scope, studies are required to consider the lifting frequencies, the effect of varying elastic strap stiffness, and monitoring joint kinematics to evaluate exosuit performance in C4 configuration for different MMH scenarios such as lifting and reaching, lifting and walking, etc.

## 7. Conclusions

The study demonstrates the importance of extended moment arm in modulating device-to-body forces. The moment arm configuration was found to affect the device-to-body forces in passive exosuit and hence the user comfort. Among the six different moment arm configurations tested, configuration C4 was found to be most beneficial in minimizing the device-to-body forces and localized discomfort without significantly affecting the trunk muscle activities while performing a symmetric lifting/lowering task with passive BASE emulator.

## Data Availability

Data sharing is not applicable to this article as no new data were created or analyzed in this study.
